# Verification of historical sketches via one-class learning on compact feature representations

**DOI:** 10.1371/journal.pone.0344796

**Published:** 2026-06-10

**Authors:** Hassan Ugail, Jan Ritch-Frel, Irina Matuzava, David G. Stork

**Affiliations:** 1 Centre for Visual Computing and Intelligent Systems, University of Bradford, Bradford, United Kingdom; 2 Independent Media Institute, New York, New York, United States of America; 3 Adjunct Professor, Stanford University, Stanford, California, United States of America; Menoufia University, EGYPT

## Abstract

Historical sketch authentication is challenging because securely attributed reference sets are often small, and stylistic evidence is carried primarily by line, texture, tonal variation, and mark-making. We present a reproducible framework for verifying historical sketches using artist-specific one-class autoencoders trained on compact handcrafted feature representations. Ten artist models were trained using authenticated sketches from six open-access cultural heritage collections. Each drawing was represented by five interpretable descriptors, namely, Fourier-domain energy, Shannon entropy, global contrast, Grey-Level Co-occurrence Matrix homogeneity, and box-counting fractal complexity. The system was evaluated using a biometric-style verification protocol in which each artist model was tested on genuine held-out works and impostor works by other artists. On the primary evaluation partition of 900 decisions, comprising 90 genuine and 810 impostor trials, the method achieved 87.6% balanced accuracy, 77.8% True Acceptance Rate, 2.6% False Acceptance Rate, 0.748 Matthews Correlation Coefficient, and 11.4% Equal Error Rate. Performance remained stable across 20 repeated random train/test splits. The proposed model also outperformed Gaussian and one-class SVM baselines, while pretrained ResNet50 and EfficientNet-V2 feature representations performed substantially worse in this data-scarce setting. Leave-one-feature-out ablation confirmed that all five descriptors contributed positively, with fractal complexity and GLCM homogeneity providing the strongest individual contributions. Error analysis revealed structured false-accept pathways to be consistent with stylistic proximity between artists. The framework provides transparent, reproducible, and interpretable quantitative evidence for historical sketch verification. It is intended to support, not replace, expert connoisseurship in attribution settings where available reference corpora are limited.

## 1. Introduction

### 1.1. Context and motivation

The authentication and attribution of historical artworks are central concerns in art history, conservation, and the art market. For works on paper, these concerns are intensified by the material and documentary conditions under which drawings survive. For example, sketchbooks may be dispersed, sheets may be trimmed or mounted, and many works exist in multiple states or workshop contexts. Connoisseurship remains indispensable in this domain, yet it is intrinsically difficult to formalise, reproduce, and quantify, particularly when disputes arise, and decision-makers require transparent evidence beyond expert opinion [1,2].

Computer vision, machine learning, and artificial intelligence offer an additional, complementary approach to analysis [3]. In particular, the logical framework of biometric verification provides an appealing analogue. For example, a test sample is verified against a target identity, and system performance is characterised by false acceptance and false rejection under explicit operating points. In art authentication, the “identity” is the target artist, and impostor trials represent non-target artists (and, in principle, forgeries). This framing falls in line with the open-set nature of attribution, where it is rarely possible to enumerate all plausible non-target classes of potential authors of the artwork in question.

Recent advances in machine learning have demonstrated promising results for image-only art attribution when large labelled datasets are available. A comprehensive review of the use of artificial intelligence in art authentication is provided by Cetinic and She (2022), who document the field’s shift from traditional computer vision methods towards deep neural networks [4]. Building on this trend, deep convolutional and attention-based architectures have been applied to artist attribution and style analysis across large corpora, achieving strong performance when abundant labelled data are available [5]. Despite these advances, such approaches generally depend on thousands of examples per artist, a requirement rarely met in the context of historical sketches or for most historical painters [3].

### 1.2. Data scarcity and the rationale for one-class verification

Deep supervised attribution methods generally require large labelled datasets and benefit from broad negative sampling [6]. Historical sketches rarely satisfy these conditions. Even for major artists, the number of authenticated drawings available as consistent digital surrogates is limited. Moreover, intra-artist variability can be substantial because sketches are often rapid, exploratory studies rather than finished works [5]. These constraints can be addressed through one-class learning, where the model learns a representation of the authentic distribution of a single artist and flags deviations as anomalous [7,8]. One-class verification is particularly appropriate when negative classes are heterogeneous, incompletely characterised, or strategically adversarial (as in forgery scenarios) [9]. This approach also fosters methodological unity and consistency, avoiding the methodological variations inherent in different analyses based on unequal choices of non-target training data.

The challenge of limited training data is not unique to art authentication. Few-shot learning approaches have been explored in various domains [10,11], but these typically still require more examples than are available for many historical artists. Transfer learning from pre-trained models offers another avenue [12], but the domain gap between natural images and historical sketches can be substantial. One-class learning sidesteps these issues by focusing solely on modelling the authentic distribution without requiring comprehensive negative examples.

### 1.3. The role of handcrafted features in data-scarce settings

Whilst end-to-end deep learning has dominated recent work in computer vision, handcrafted features retain important advantages in data-scarce scenarios. They reduce sample complexity through dimensionality reduction, provide interpretability enabling expert validation, incorporate domain knowledge, and offer greater robustness to distribution shift than learnt representations when training sets are small [5,12]. For sketch authentication, colour information is limited, and style is expressed primarily through marks, shading, and tonal distribution. Carefully designed handcrafted features can capture essential stylistic signals whilst remaining trainable with minimal data.

Texture features derived from Grey-Level Co-occurrence Matrices have proven effective for distinguishing artistic techniques [13]. Frequency domain analysis reveals characteristic rhythmic patterns in artists’ marks [14]. Fractal analysis captures the hierarchical complexity of mark-making [15–17]. Information-theoretic measures quantify tonal complexity and distributional properties [18,19]. By combining features that have been proven to be informative in many domains (including art analysis), we can construct compact yet informative representations suitable for one-class learning.

### 1.4. Scope and contributions

This study develops a reproducible verification framework for sketch authentication under severe corpus size constraints. The central methodological contribution is an artist-specific one-class autoencoder verifier trained on interpretable handcrafted features well suited to line-dominant media. The empirical contribution is a multi-artist evaluation across ten historical artists, reporting both pooled and artist-specific biometric metrics with Wilson confidence intervals, partition robustness evidence from repeated random sub-sampling, a controlled leave-one-feature-out ablation, and a structured attribution of false-accept pathways to identify systematic confusability between artists.

Our principal contributions are as follows:

- A novel application of one-class autoencoder architecture to historical sketch authentication, demonstrating effective discrimination despite severe data scarcity (20 training images per artist).
- Identification and formal definition of five literature-motivated handcrafted features—Fourier energy, Shannon entropy, contrast, GLCM homogeneity, and box-counting fractal dimension—selected to capture distinct properties of artistic style in line-dominant media, with feature necessity confirmed empirically by ablation.
- Comprehensive multi-artist evaluation using a rigorous biometric verification framework with 900 trials, reporting all metrics with Wilson binomial confidence intervals appropriate for small sample sizes, and using MCC and balanced accuracy as primary discrimination summaries to account for class imbalance.
- Partition robustness analysis via 20 independent repeated random sub-samplings (seeds 0–19) of the 29-image corpus per artist, establishing that the primary reported results are representative rather than an artefact of a single train/test split.
- A systematic leave-one-feature-out ablation study using a fixed-capacity architecture across all conditions to isolate feature contribution from model-capacity effects, identifying fractal dimension and GLCM homogeneity as the most informative individual features.
- Pairwise confusion attribution revealing structured error relations consistent with art-historically interpretable stylistic proximity.

## 2. Related work

### 2.1 Machine learning in art authentication

Research on computational art analysis spans traditional feature engineering, modern deep learning, and hybrid approaches. Surveys in computer vision applied to art have highlighted both the promise of machine learning for attribution and the practical barriers posed by limited data, domain shift, and the interpretability gap between learnt features and art-historical concepts [1,2]. Early wavelet analysis applied to paintings demonstrated that computational methods could detect stylistic patterns not readily apparent to human observers [20]. Likewise, sets of oriented spatial frequency filters sufficed for state-of-the-art accuracy in image-only authentication of Jackson Pollock’ s drip paintings [21].

Recent advances in deep learning have shown impressive results when sufficient image data are available. Castellano and Vessio provide a comprehensive overview of deep learning approaches to pattern extraction in paintings and drawings, documenting accuracies exceeding 90% on large-scale datasets and showing how convolutional and attention-based architectures capture long-range stylistic dependencies [5]. The broader landscape of artificial intelligence in art has been surveyed by Cetinic and She, who identify three major approaches. They are supervised classification using convolutional neural networks, transfer learning from pre-trained models, and generative modelling for anomaly detection [4]. More recently, vision transformer architectures have been applied to art classification, achieving strong results on curated datasets with sufficient training examples per class [22]. However, such models are particularly data-hungry due to their lack of inductive spatial bias, making them unsuitable for the severely data-scarce settings addressed here.

However, these impressive results typically require large corpora of training data. Large-scale art datasets containing thousands of images per artist category have been curated specifically to enable the training of deep networks [23]. The data efficiency challenge has been addressed explicitly through few-shot learning approaches capable of learning from as few as five examples per class via meta-learning, though performance remained below that achieved with larger datasets [10,11]. Transfer learning has also been investigated for art classification, demonstrating that pre-trained features can partially address data scarcity, yet substantial performance gaps compared to in-domain training persist [12].

For specific authentication challenges, targeted approaches have proven effective. Methods accounting for contextual information have been developed for painting classification and retrieval [24], whilst deep networks with data augmentation have been applied to forgery detection [25]. Techniques originally developed for computer-generated image detection have also been adapted for art authentication [14]. Notably, most existing work has focused on oil paintings, with limited attention to works on paper, motivating the current study’s focus on sketch authentication.

### 2.2. Autoencoders and anomaly detection

In parallel, the anomaly detection literature has formalised one-class learning and reconstruction-based scoring, with autoencoders remaining a standard approach when the objective is to model “normal” data and detect deviations [7,8]. Originally introduced by Rumelhart et al. and refined over subsequent decades [26], autoencoders learn to compress input data into lower-dimensional latent representations and then reconstruct the original input. By training exclusively on representative data from a single class, the autoencoder becomes specialised at reconstructing similar patterns; anomalous data leads to higher reconstruction errors, and these provide a quantitative anomaly score.

A comprehensive survey of deep learning approaches to anomaly detection identifies autoencoders as particularly suitable for one-class learning scenarios where normal data are abundant but anomalous data are scarce or unknown [7]. That taxonomy distinguished between reconstruction-based methods, which use reconstruction error as an anomaly score, and embedding-based methods, which learn compact representations optimised for separating normal from anomalous data. An extensive complementary review highlights advances in convolutional autoencoders, variational autoencoders, and adversarial training approaches, noting that reconstruction-based methods remain effective for high-dimensional data such as images [8].

Recent work has addressed training stability and performance optimisation through several architectural innovations. Gong et al. introduced memory-augmented autoencoders that explicitly store prototypical normal patterns to sharpen anomaly discrimination [27], whilst deep one-class classification methods that constrain the latent space to a compact hypersphere have been developed to ensure more discriminative representations [28]. Building on memory-based architectures, Park et al. extended this approach with compactness and separateness losses applied to memory items, boosting discriminative power and demonstrating improved performance on standard anomaly detection benchmarks [29].

In medical imaging—a domain that shares important parallels with art authentication in its reliance on small corpora of abnormal examples and high-dimensional data—autoencoders have proven highly effective. Deep autoencoder models applied to brain MRI analysis have achieved state-of-the-art anomaly detection for identifying pathological changes [30], and generative adversarial network-based approaches have demonstrated strong performance in retinal imaging [31]. Particularly relevant to the present work, a combination of texture features with deep learning for breast cancer classification achieved high accuracy by leveraging handcrafted features alongside learnt representations [32]—a hybrid strategy that directly influenced our choice of methodology.

### 2.3. Feature engineering for art analysis

Throughout a range of domains, pattern classification accuracy generally depends upon preprocessing and the choice of visual features to be extracted, and the domain of art drawings is no exception. Sketches provide less colour information than paintings and often express style through mark-making, tonal distribution, and compositional density. Consequently, the use of interpretable features derived from frequency analysis, information theory, and texture statistics improve classification performance in data-scarce settings because they reduce dimensionality whilst retaining meaningful stylistic signal [33].

Texture features derived from Grey-Level Co-occurrence Matrices (GLCM) remain widely used in image analysis. Originally introduced for image classification, GLCM-based measures—including contrast, homogeneity, energy, and entropy—capture statistical properties of spatial relationships between pixel intensities, with homogeneity and entropy identified as particularly discriminative for characterising spatial structure [13].

Frequency domain analysis continues to provide insights into artistic style. Wavelet and Fourier analysis applied to forgery detection has demonstrated that authentic works exhibit characteristic frequency energy distributions—reflecting the scale and regularity of mark-making—that vary systematically across artistic traditions and that forgeries struggle to replicate [14,17]. For these reasons, we included Fourier energy as a discriminative feature.

Fractal analysis has evolved beyond simple dimension estimation to become a tool for characterising artistic complexity in some domains. The theoretical foundations of fractal geometry, established by Mandelbrot [15], underpin a body of applied work in art analysis. Refined fractal methods applied to Jackson Pollock authentication have demonstrated discrimination between authentic drip paintings and imitations, addressing criticisms of earlier approaches [16]. The use of orientation-tuned spatial frequency filters proved superior to such simple fractal features of authenticating Pollock’ s drip paintings [21]. Multifractal analysis applied across art history suggests that fractal complexity may have evolved systematically over centuries and differs between artistic movements [17].

Information-theoretic features have gained renewed attention for art analysis. Building on the foundational framework of Shannon [18], entropy-based measures applied to artworks have demonstrated that entropy captures perceptually meaningful aspects of artistic composition [19]. Investigations into aesthetic preference have further found that moderate complexity and entropy correlate with aesthetic appeal [34], reinforcing the perceptual relevance of such measures for characterising artistic style.

Feature fusion approaches have shown that combining descriptors from different sources yields richer representations than any single feature type alone [35]. This finding directly informed our selection of five distinct features operating at different scales and capturing different properties of artistic style. The present paper adopts a compact feature vector designed to capture multiple aspects of drawing structure and complexity whilst remaining interpretable to domain experts.

### 2.4. Biometric verification frameworks

Art authentication shares methodological parallels with biometric verification, where individuals are authenticated based on intrinsic characteristics. The biometric literature provides rigorous frameworks for evaluating authentication systems that translate naturally to the domain of art verification. A comprehensive introduction to biometric systems, defining standard metrics including False Acceptance Rate (FAR), False Rejection Rate (FRR), and Equal Error Rate (EER) has been provided by Jain et al. [36], whose framework for evaluating one-to-many identification scenarios directly applies to art authentication, where a piece of work is compared against a database of known artists.

Evaluation methodologies for biometric systems under realistic conditions have emphasised the importance of appropriate confidence intervals when sample sizes are limited [37]. The advocacy for Wilson binomial intervals [38,39] over normal approximations influenced our statistical approach. Challenges in comparing biometric systems across different datasets and evaluation protocols, including the need for standardised reporting of performance metrics, have been addressed in large-scale face recognition studies [40].

The concept of impostor trials in biometric verification directly parallels the challenge of distinguishing an artist’s genuine works from those by other artists. An overview of presentation attack detection addressing the problem of deliberate spoofing—analogous to forgery in art authentication—is provided by Marcel et al. [41], whose discussion of anomaly-based detection methods informed our one-class learning approach. Thus, the biometric framework’s explicit treatment of operating points, trade-offs between false acceptance and false rejection, and evaluation under realistic trial structures provides a solid foundation for art authentication research.

## 3. Materials and methods

### 3.1 Ethics statement

This study did not involve human subjects, animal experimentation, or the collection of personal data. All images were obtained from publicly available open-access repositories operated by major cultural institutions, used in accordance with their respective open-access licensing policies. No institutional ethics approval was required for this study.

### 3.2. Dataset and curation

We curated a dataset of *K* = 10 artists. For each artist, *n*_train_ = 20 authenticated works were used for model training and *n*_test_ = 9 authenticated works were reserved for evaluation, yielding $|S_{a}|=29$ images per artist and 290 images in total. Training and test sets are strictly disjoint for every artist in every experiment. No test image is used at any stage of model training, feature standardisation, or threshold calibration. The evaluation protocol comprises 9 genuine trials and 81 impostor trials per artist-specific model (9 test images from each of the nine non-target artists), yielding 900 pooled verification decisions across the ten models. To establish that performance metrics are not sensitive to the particular partition chosen, a repeated random sub-sampling analysis over 20 independent train/test splits is reported in Section 3.8 (Methods) and Section 4.8 (Results).

Images were sourced from publicly available, open-access collections, including the Metropolitan Museum of Art’s online collection, the Ashmolean Collections Catalogue, the Morgan Library and Museum, the Royal Collection Trust (UK), the Victoria and Albert Museum Collections, and the Casa Buonarroti online catalogue. Selection was restricted to drawings and sketches attributed to ten artists:

- Anthonis van den Wijngaerde (c. 1510–1561, Flemish topographical artist), sourced from the Metropolitan Museum of Art and the Ashmolean Collections Catalogue.
- John Constable (1776–1837, English landscape painter), sourced from the Metropolitan Museum of Art and the Victoria and Albert Museum Collections.
- Giovanni Francesco Barbieri, also known as Guercino (1591–1666, Italian Baroque painter), sourced from the Metropolitan Museum of Art and the Ashmolean Collections Catalogue.
- John William Waterhouse (1849–1917, English Pre-Raphaelite painter), sourced from the Metropolitan Museum of Art and the Victoria and Albert Museum Collections.
- Michelangelo Buonarroti (1475–1564, Italian Renaissance master), sourced from the Metropolitan Museum of Art, the Morgan Library and Museum, the Royal Collection Trust (UK), and the Casa Buonarroti catalogue.
- Raffaello Sanzio, known as Raphael (1483–1520, Italian Renaissance master), sourced from the Metropolitan Museum of Art and the Ashmolean Collections Catalogue.
- Thomas Sully (1783–1872, American portrait painter), sourced from the Metropolitan Museum of Art.
- William Trost Richards (1833–1905, American landscape painter), sourced from the Metropolitan Museum of Art.
- James McNeill Whistler (1834–1903, American tonalist painter), sourced from the Metropolitan Museum of Art.
- Wilhelm Stettler (1643–1708, Swiss draughtsman), sourced from the Metropolitan Museum of Art and the Ashmolean Collections Catalogue.

Our selection criteria aimed to minimise confounds, namely, images were chosen to avoid palimpsests or multi-work sheets where possible, and were cropped to reduce borders and extraneous page context, thereby limiting the influence of mount tone, margins, and institutional photographing conventions. Note, such confounds have plagued analogous studies where, for instance, a meaningless background colour determined the automatic recognition of works. Artworks were selected from fully provenanced collections where attribution is supported by unchallenged scholarship and, where available, documentary evidence. The temporal and stylistic diversity—spanning Renaissance through the nineteenth century, and including Italian, Flemish, English, American, and Swiss traditions—ensures that the comparison set presents a genuine challenge for authentication.

### 3.3. Preprocessing

Each image was resized to 224 × 224 pixels using bicubic interpolation, which provides smooth interpolation appropriate for natural images and artwork. For features defined on luminance or texture statistics, images are converted to greyscale using the standard luminance transform,


$$\begin{array}{c} Y=0.299R+0.587G+0.114B, \end{array}$$ (1)

which approximates human luminance perception by weighting the green channel most heavily. Intensities are normalised to [0,1] by dividing by 255, ensuring consistent numeric ranges across features. This standardised preprocessing pipeline ensures that features are computed consistently across all images regardless of original resolution, colour depth, or digitisation protocols.

### 3.4. Feature extraction

For each image *i*, we compute a five-dimensional feature vector,


$$\begin{array}{c} {𝐟}_{i}=[E_{\text{Fourier},i},\;H_{\text{Shannon},i},\;\sigma_{\text{contrast},i},\;H_{\text{homogeneity},i},\;D_{\text{BC},i}{]}^{⊤}\in {\mathbb{R}}^{5}, \end{array}$$ (2)

where *D*_BC_ denotes a box-counting estimate of fractal complexity (the Hausdorff-Besicovitch dimension). The features are chosen to capture distinct aspects of sketches, namely, global frequency energy reflecting mark-making scale, tonal information content measuring distributional complexity, intensity dispersion quantifying value range, local spatial regularity characterising texture smoothness, and multi-scale edge complexity encoding hierarchical structure. These features are literature-motivated and operate at different scales, providing a compact yet informative representation. The discriminative contribution of each feature is empirically validated through a systematic leave-one-feature-out ablation analysis reported in Section 3.9 (Methods) and Section 4.9 (Results).

Although one could incorporate additional features, for instance, based on statistics of mark lengths, widths, curvatures, topological measures, and so on, we found our present set sufficiently informative for accurate classification tasks. Additional features would increase the overall complexity and computational cost of our system, and likely increase the risk of overfitting.

To reduce sensitivity to digitisation variability, all images are cropped to the artwork region during curation to remove page margins and mounts. No additional background masking is applied beyond this crop. Images are resized to 224 × 224 and normalised to [0,1] prior to feature computation.

For GLCM features, greyscale intensities are uniformly quantised to *L*_*q*_ = 64 levels prior to GLCM computation. Homogeneity is computed using distance *d* = 1 pixel over orientations $\theta \in \{0^{\circ },45^{\circ },90^{\circ },135^{\circ }\}$ and averaged to provide approximate rotation invariance.

For the box-counting fractal dimension estimate, edges are extracted using Canny edge detection with Gaussian smoothing σ=1.0 and hysteresis thresholds *t*_low_ = 0.10 and *t*_high_ = 0.20 (defined on the [0,1] intensity scale). Box counts are computed over box sizes ε∈{2,4,8,16,32,64} pixels, and the fractal dimension is estimated as the slope of a least-squares regression of logN(ε) on log(1/ε).

#### 3.4.1. Fourier Energy, *E*_*Fourier*_.

Let *P*(*i*,*j*) denote the greyscale image of size *M* × *N*. The two-dimensional Discrete Fourier Transform (DFT) decomposes the image into frequency components,


$$\begin{array}{c} F(u,v)=\sum_{i=0}^{M-1}\sum_{j=0}^{N-1}P(i,j)\exp\left[-2\pi i\left(\frac{ui}{M}+\frac{vj}{N}\right)\right], \end{array}$$ (3)

where u∈{0,1,…,M−1} and v∈{0,1,…,N−1} are frequency indices, and $i=\sqrt{-1}$. The magnitude spectrum is,


$$\begin{array}{c} |F(u,v)|=\sqrt{ℜ(F(u,v){)}^{2}+ℑ(F(u,v){)}^{2}}. \end{array}$$ (4)

To avoid sensitivity to overall image brightness—since by Parseval’s theorem the total energy $\sum_{u,v}|F(u,v){|}^{2}$ equals the sum of squared pixel intensities and is therefore dominated by the DC component *F*(0,0)—we exclude the DC term and compute energy over the non-zero-frequency components only, such that,


$$\begin{array}{c} E_{\text{Fourier}}=\sum_{\begin{array}{c} u=0\\ \left(u,v)\neq (0,0\right) \end{array}}^{M-1}\sum_{v=0}^{N-1}{\left|F(u,v)\right|}^{2}. \end{array}$$ (5)

This AC energy statistic reflects the distribution of signal energy across spatial frequencies independently of mean brightness, and is sensitive to the prevalence of fine mark-making versus broader tonal masses. Artists with finer, more detailed mark-making tend to concentrate energy in higher frequencies, whilst those favouring softer, broader strokes concentrate energy in lower frequencies [14]. Since all images are normalised to [0,1] prior to feature computation, the AC energy remains comparable across images with different original brightness levels.

#### 3.4.2. Shannon entropy, *H*_*Shannon*_.

Given a 256-bin histogram of greyscale intensities, with probabilities p(k)=h(k)/(M·N) where *h*(*k*) is the count in bin *k*, the Shannon entropy [18] is,


$$\begin{array}{c} H_{\text{Shannon}}=-\sum_{k=0}^{255}p(k)\log_{2}p(k), \end{array}$$ (6)

with the convention $0\log_{2}0=0$. Entropy is the maximum ($\log_{2}256=8$ bits) for a uniform distribution where all intensity values are equally probable, and minimum (0 bits) for a constant image. This feature approximates tonal complexity and distributional spread of values, capturing the diversity and unpredictability of intensity patterns. Artists who create smooth gradations and limited tonal ranges produce lower entropy, whilst those employing varied, complex tonal structures produce higher entropy [19].

#### 3.4.3. Contrast, $\sigma_{contrast}$.

Global contrast is measured as the standard deviation of pixel intensities, quantifying the spread of values around the mean,


$$\begin{array}{c} \sigma_{\text{contrast}}=\sqrt{\frac{1}{MN}\sum_{i=0}^{M-1}\sum_{j=0}^{N-1}{\left(P(i,j)-\mu \right)}^{2}}, \end{array}$$ (7)

where $\mu =\frac{1}{MN}\sum_{i=0}^{M-1}\sum_{j=0}^{N-1}P(i,j)$ is the mean intensity. Contrast relates to the tonal range employed by the artist. Artists who work with strong value contrasts, such as dramatic chiaroscuro, produce high contrast measures, whilst those who work within a narrow tonal range produce lower contrast. This simple but informative statistic captures fundamental choices about value structure.

#### 3.4.4. GLCM homogeneity, *H*_*homogeneity*_.

A Grey-Level Co-occurrence Matrix (GLCM) captures local spatial relationships between quantised intensities [13]. The GLCM records how frequently pairs of pixels with specific intensity values occur at a specified spatial relationship. To improve robustness and reduce sparsity, intensities are quantised to *L*_*q*_ = 64 levels prior to GLCM computation. For distance *d* = 1 pixel and orientations $\theta \in \{0^{\circ },45^{\circ },90^{\circ },135^{\circ }\}$, we compute a normalised, symmetric GLCM $G_{d,\theta }(i,j)$ and define homogeneity as,


$$\begin{array}{c} H_{\text{homogeneity}}=\frac{1}{4}\sum_{\theta }\sum_{i=0}^{L_{q}-1}\sum_{j=0}^{L_{q}-1}\frac{G_{d,\theta }(i,j)}{1+|i-j|}. \end{array}$$ (8)

This measure is high when the GLCM has high values along its diagonal (indicating similar adjacent pixels) and low when values are spread away from the diagonal (indicating dissimilar adjacent pixels). Homogeneity reflects the smoothness or uniformity of texture. Artists who blend and smooth their marks create high homogeneity, whilst those who juxtapose contrasting marks create lower homogeneity. Averaging across four orientations provides approximate rotation invariance [13].

#### 3.4.5. Fractal complexity via Box-counting, *D*_*BC*_.

We compute a box-counting fractal dimension estimate (often used as a practical proxy for Hausdorff-type scaling behaviour) by applying Canny edge detection followed by multi-scale box counting and log-log regression. Edges are extracted using a Canny detector with fixed parameters to form a binary edge map. The box-counting procedure [15] evaluates the number N(ε) of boxes of side length ε required to cover edge pixels. This is computed over box sizes ε∈{2,4,8,16,32,64} pixels. The estimated box-counting dimension is,


$$\begin{array}{c} D_{\text{BC}}=slope\left(\log N(\varepsilon )\text{ vs. }\log(1/\varepsilon )\right). \end{array}$$ (9)

The fractal dimension captures how the apparent complexity of the edge structure scales with the resolution of observation. Sketches with intricate, self-similar mark-making (such as dense crosshatching or complex foliage) exhibit higher fractal dimensions approaching 2, whilst those with simpler, more uniform line work exhibit lower dimensions closer to 1. This measure has proven effective for characterising artistic complexity and has been applied to art authentication problems [16,17].

#### 3.4.6. Feature standardisation.

Because features operate on different scales and have different physical units (energy in arbitrary units, entropy in bits, contrast in intensity units, homogeneity dimensionless, fractal dimension dimensionless), *z*-score standardisation is applied using statistics computed on the training set for each artist,


$$\begin{array}{c} {\tilde{f}}_{ij}=\frac{f_{ij}-\mu_{j}}{\sigma_{j}}, \end{array}$$ (10)

where $\mu_{j}$ and $\sigma_{j}$ are the mean and standard deviation for feature *j* computed over the twenty training images. This standardisation, much like a whitening transform, ensures that no single feature dominates the learning process due to its numeric scale, and that the autoencoder treats each feature dimension with comparable importance. Of course, through learning, different features (components in the vector of Eq. 2) will be weighted differently for optimal classification performance. The same normalisation parameters are applied consistently to test images; no test-set information is used in their computation.

### 3.5. One-class autoencoder verifier

#### 3.5.1. Model definition and architecture.

Each artist is assigned an independent one-class verifier implemented as a feedforward autoencoder operating on the five-dimensional standardised feature vector. Let $𝐱\in {\mathbb{R}}^{5}$ denote an input feature vector from Eq. 2. The encoder maps **x** to a latent representation $𝐡\in {\mathbb{R}}^{k}$ (where *k* < 5) via,


$$\begin{array}{c} 𝐡=f(𝐱;\theta_{e}), \end{array}$$ (11)

and the decoder reconstructs $\hat{𝐱}$ via,


$$\begin{array}{c} \hat{𝐱}=g(𝐡;\theta_{d}), \end{array}$$ (12)

where $\theta_{e}$ and $\theta_{d}$ represent the encoder and decoder parameters, respectively. The autoencoder learns a compressed representation that captures the essential structure of the authentic feature distribution.

The model is trained to minimise the mean squared reconstruction error over authenticated training samples $𝒟=\{{𝐱}_{1},{𝐱}_{2},\ldots ,{𝐱}_{N}\}$ where *N* = 20 for each artist,


$$\begin{array}{c} ℒ(\theta_{e},\theta_{d})=\frac{1}{N}\sum_{i=1}^{N}\|{𝐱}_{i}-{\hat{𝐱}}_{i}\overset{2}{\underset{2}{\|}}. \end{array}$$ (13)

**Fig 1:**
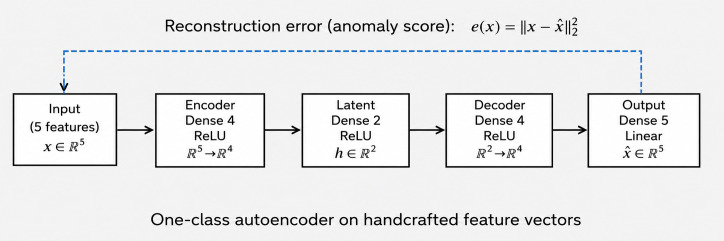
Feedforward autoencoder architecture for one-class sketch verification. The full five-feature model uses layers 5→4→2→4→5; the fixed-capacity ablation model uses the same architecture with the omitted feature’s standardised dimension set to zero. ReLU activations are used in all hidden layers, and the output layer uses a linear activation. The bottleneck dimension of *k* = 2 forces the model to learn a compact latent representation of each artist’s authentic feature distribution. The reconstruction error $e(𝐱)=\|𝐱-\hat{𝐱}\overset{2}{\underset{2}{\|}}$ serves as the anomaly score for verification.

The architecture is deliberately compact to match the dimensionality and data regime. The full five-feature model uses a symmetric bottleneck network with layers 5→4→2→4→5 nodes, using rectified linear unit (ReLU) activations in hidden layers and a linear output layer (Fig 1). This shallow architecture with limited capacity is appropriate given the small training set size, reducing the risk of overfitting whilst maintaining sufficient representational power. The bottleneck dimension of *k* = 2 forces the model to learn a compact latent representation that captures only the most essential aspects of the feature distribution. The axes of this highly compressed latent space are unlikely to correspond directly to art-historical properties. Nevertheless, they represent nonlinear mixtures of the five input features as in Eq. 2.

Training is conducted using the Adam optimiser [42], an adaptive learning rate method that combines the benefits of AdaGrad and RMSProp. A learning rate of γ=0.001 is used with batch processing of the full training set (given the small sample size). Training proceeds for a fixed maximum of 500 epochs, which was observed to produce stable reconstruction loss in all runs across all ten artist models. A fixed random seed (seed  =  42) is used for all autoencoder weight initialisations and training runs to ensure reproducibility. All hyperparameters (architecture depth, bottleneck dimension, learning rate, number of epochs, and threshold quantile) were fixed *a priori* based on the data dimensionality and regime size, and were not tuned on test data. The threshold quantile *q* = 0.95 was selected based on domain considerations (Section 4.5) rather than optimisation on held-out performance.

#### 3.5.2. Anomaly score and decision rule.

For a test sample **x**, the anomaly score is the reconstruction error,


$$\begin{array}{c} e(𝐱)=\|𝐱-\hat{𝐱}\overset{2}{\underset{2}{\|}}, \end{array}$$ (14)

where $\hat{𝐱}=g(f(𝐱;\overset{*}{\underset{e}{\theta }});\overset{*}{\underset{d}{\theta }})$ and $\overset{*}{\underset{e}{\theta }},\overset{*}{\underset{d}{\theta }}$ are the optimised parameters after training. High reconstruction errors indicate that the test sample differs significantly from the training distribution, suggesting possible misattribution or forgery.

Verification requires converting this continuous anomaly score into a hard binary decision. For each artist-specific verifier, the operating threshold $\tau_{a}$ is determined by 5-fold cross-validation on the *n*_train_ = 20 training works. In each fold, four fifths of the training set (16 images) are used to train a candidate autoencoder, and the held-out fifth (4 images) provides reconstruction errors on unseen training-distribution samples. The per-fold errors from held-out images are pooled across all five folds to form a distribution of out-of-fold reconstruction errors $\{e_{cv,i}\overset{20}{\underset{i=1}{\}}}$, and the threshold is set as $\tau_{a}=Q_{q}(\{e_{cv,i}\})$, where $Q_{q}(\cdot )$ denotes the empirical quantile at level *q*. The final model used for evaluation is then retrained on all 20 training images with the same architecture and hyperparameters, and $\tau_{a}$ is applied directly to this final model. A test sample is accepted as genuine if $e(𝐱)\leq \tau_{a}$ and rejected otherwise.

This cross-validated threshold calibration ensures that the threshold reflects reconstruction errors on samples not used to fit the model weights in each fold. One subtlety of this procedure is that $\tau_{a}$ is derived from five candidate models each trained on 16-image subsets, and is then applied to the final model retrained on all 20 images. Because the final model has access to more training data, its reconstruction errors on training-distribution samples may differ systematically from those of the 16-image CV fold models; this transfer from CV models to the final model is an approximation rather than an exact calibration. In practice, this effect is expected to be small given the shallow architecture and the small increment in training set size (16–20 images), but it is noted as a limitation in Section 5.

In all reported experiments, we use *q* = 0.95, selected *a priori* based on the authentication use case, i.e., incorrectly admitting a non-genuine work may carry legal, financial, and reputational consequences, whereas flagging a target work for further expert review is a far less costly error. This operating point is therefore chosen to prioritise low false acceptance under open-set conditions [37]. The sensitivity analysis in Section 4.5 confirms that the main conclusions are stable across neighbouring operating points. No test-set information is used at any stage of threshold determination. The complete pipeline is formalised in Algorithm 1.

**Algorithm 1** Primary evaluation pipeline for artist authentication in historical drawings

**Inputs:**  $A=\{a_{1},\ldots ,a_{K}\}$ (*K* = 10); authenticated image sources 𝒞; *n*_train_ = 20; *n*_test_ = 9; *q* = 0.95; *V* = 5 (CV folds); autoencoder seed  =  42.

**Outputs:** Per-artist and pooled decisions; confusion matrices; FAR/FRR/TAR, MCC, balanced accuracy, EER; Wilson CIs.

**foreach** a∈A **do**

  Curate and crop images for *a*; quality control; select *S*_*a*_ with $|S_{a}|=n_{train}+n_{test}$;

  Preprocess (resize 224 × 224, greyscale, normalise);

  Compute $𝐟(G)\in {\mathbb{R}}^{5}$ (Fourier energy, entropy, contrast, GLCM homogeneity, fractal dimension);

  Split into Train_*a*_ (fixed primary partition) and Test_*a*_;

  Compute $\left(\mu_{a},\sigma_{a}\right)$ from Train_*a*_; standardise all images;

  Train AE_*a*_ (5→4→2→4→5) on Train_*a*_ with fixed seed;

  **5-fold CV threshold:** pool out-of-fold reconstruction errors; set $\tau_{a}=Q_{q}(\{e_{cv,i}\})$;

  Evaluate: accept probe iff $e(𝐱)\leq \tau_{a}$; compute TP/FN/FP/TN across genuine and impostor trials;

Compute per-artist and pooled metrics; report Wilson CIs; compute EER by linear interpolation of FAR/FRR curves;

**Algorithm 2** Partition robustness (RSS) and leave-one-feature-out (LOFO) ablation

**Inputs:**  All 290 images (29 per artist); *R* = 20 RSS seeds (0–19); *q* = 0.95; fixed AE seed  =  42.

**Outputs:** RSS: mean/SD/range of pooled FAR and TAR over *R* draws; per-artist mean FAR across draws. LOFO: pooled FAR, TAR, MCC, ΔMCC for each omitted feature.

// Part A: Partition Robustness (RSS)

**for** r←0 **to** *R* − 1 **do**

  For each artist *a*, randomly assign 20 images to $\overset{\left(r\right)}{\underset{a}{Train}}$ and 9 to $\overset{\left(r\right)}{\underset{a}{Test}}$ using seed *r;*

  Run Algorithm 1 with this partition (autoencoder seed  =  42 throughout);

  Record pooled FAR^(*r*)^ and TAR^(*r*)^, and per-artist FARa(r);

Report mean, SD, min, max of pooled FAR and TAR; report per-artist mean FAR across draws;

// Part B: LOFO Ablation

**for** *each feature* $f_{j}\in \{E_{\text{Fourier}},H_{\text{Shannon}},\sigma_{\text{contrast}},H_{\text{homogeneity}},D_{\text{BC}}\}$

**do**

  Construct ablated vector ${\tilde{𝐟}}^{\left(-j\right)}\in {\mathbb{R}}^{5}$: standardise normally, then set standardised dimension *j* to zero;

  Run Algorithm 1 on fixed primary partition with ${\tilde{𝐟}}^{\left(-j\right)}$ and identical 5→4→2→4→5 architecture;

  Record pooled FAR, TAR, MCC; compute ΔMCC relative to full five-feature model;

### 3.6. Verification framework and metrics

Each artist-specific autoencoder functions as a binary verifier under a standard biometric evaluation protocol [36]. For each artist *a*, a dedicated verifier is trained using only the *n*_train_ = 20 authenticated training works of that artist. Evaluation uses *n*_test_ = 9 held-out authenticated works per artist. For a given verifier targeting artist *a*, *genuine trials* consist of the 9 test works by *a*, whilst *impostor trials* consist of the 9 test works from each of the remaining *K* − 1 = 9 artists, yielding 9 × 9 = 81 impostor trials per verifier. Across all *K* = 10 verifiers, this produces 90 genuine trials and 810 impostor trials, for 900 verification decisions in total.

Outcomes are defined according to standard conventions. True Accept (TP) occurs when a genuine image is correctly classified as genuine. False Reject (FN) occurs when a genuine image is incorrectly classified as an impostor. False Accept (FP) occurs when an impostor image is incorrectly classified as genuine. True Reject (TN) occurs when an impostor image is correctly classified as an impostor. These outcomes form the 2 × 2 confusion matrix from which all performance metrics are derived.

Performance is summarised with biometric metrics appropriate for verification systems. The False Acceptance Rate measures the proportion of impostor trials incorrectly accepted: $FAR=FP/N_{\text{impostor}}$, where *N*_impostor_ = 81 per artist model. The False Rejection Rate measures the proportion of genuine trials incorrectly rejected: $FRR=FN/N_{\text{genuine}}$, where *N*_genuine_ = 9 per artist model. The True Acceptance Rate (also called Genuine Acceptance Rate) is $TAR=1-FRR=TP/N_{\text{genuine}}$. Specificity (True Rejection Rate) is $TN/N_{\text{impostor}}$. The Equal Error Rate (EER) is the operating point at which FAR  =   FRR and is computed by linear interpolation of the FAR–FRR curve across threshold values; it provides a single-number, threshold-independent summary of system discrimination.

The trial structure yields a 9:1 impostor-to-genuine imbalance, which inflates overall accuracy as a summary metric. Accordingly, the primary summary metrics throughout are the Matthews Correlation Coefficient (MCC) [43] and balanced accuracy, both of which account for class imbalance. Overall accuracy is reported for completeness but is not used as the primary discrimination measure. MCC is defined as,


$$\begin{array}{c} MCC=\frac{TP\cdot TN-FP\cdot FN}{\sqrt{\left(TP+FP)(TP+FN)(TN+FP)(TN+FN\right)}}, \end{array}$$ (15)

and balanced accuracy is,


$$\begin{array}{c} Balanced\;Accuracy=\frac{1}{2}\;\left(\frac{TP}{TP+FN}+\frac{TN}{TN+FP}\right). \end{array}$$

MCC ranges from −1 (total disagreement) through 0 (no better than random) to +1 (perfect prediction).

### 3.7. Confidence intervals

Because genuine trials per model are small (*n* = 9), and binomial rates near 0 or 1 are common (particularly for FAR), uncertainty is quantified using Wilson binomial confidence intervals [38], which offer more reliable coverage than normal approximations in small-sample settings [37,39]. For an observed proportion $\hat{p}=x/n$ where *x* is the number of successes in *n* trials, and using *z* = 1.96 for 95% confidence, the Wilson interval is,


$$\begin{array}{c} \frac{\hat{p}+z^{2}/(2n)\pm z\sqrt{\hat{p}(1-\hat{p})/n+z^{2}/(4n^{2})}}{1+z^{2}/n}. \end{array}$$ (16)

This interval has better coverage properties than the Wald (normal approximation) interval, particularly when *n* is small or $\hat{p}$ is near the boundaries. All reported confidence intervals use this method.

### 3.8. Partition robustness via repeated random sub-sampling

Because the primary evaluation rests on a single fixed partition of 9 test images per artist drawn from 29 available images, we assess the stability of reported performance through repeated random sub-sampling (RSS). For each of *R* = 20 independent random draws (seeds 0–19 inclusive), a new stratified train/test split is constructed by sampling without replacement, i.e., for each artist, 20 images are randomly assigned to training and the remaining 9 to evaluation. The full pipeline—feature standardisation, autoencoder training (autoencoder weight seed  =  42 throughout all draws), 5-fold CV threshold calibration (*q* = 0.95), and biometric evaluation—is re-executed independently for each split, yielding 20 pooled FAR and TAR estimates under the identical protocol used for the primary results.

The choice of *R* = 20 provides a standard error of approximately $\text{SD}/\sqrt{20}$ for the mean of each metric; convergence of the running mean and standard deviation is confirmed to be stable by drawing 15 in practice. This procedure does not constitute cross-validation in the strict sense, since each draw produces an independent model and evaluation rather than a held-out estimate from a shared model; its purpose is to characterise the variability of the reported metrics across plausible partitions of the available data. Summary statistics (mean, standard deviation, minimum, maximum) are reported for pooled TAR and FAR, and per-artist mean FAR across the 20 draws is reported to confirm that artist-level rankings are stable. Consistency between the primary partition result and the RSS distribution confirms that the reported performance is representative of the framework’s population-level behaviour rather than sensitive to the particular partition chosen. The RSS procedure is formalised in Algorithm 2 (Part A).

### 3.9. Leave-one-feature-out ablation study

To assess the contribution of each individual feature to system performance and to verify that all five components provide discriminative information, we conduct a leave-one-feature-out (LOFO) ablation analysis using a *fixed-capacity* architecture across all conditions. For each of the five features $f_{j}\in \{E_{\text{Fourier}},H_{\text{Shannon}},\sigma_{\text{contrast}},H_{\text{homogeneity}},D_{\text{BC}}\}$, an ablated five-dimensional feature vector is constructed by standardising all five features normally and then setting standardised dimension *j* to zero in the model input; this is equivalent to imputing the training-set mean for the omitted feature. The identical 5→4→2→4→5 architecture is retained for all conditions. This zero-padding approach—rather than removing the dimension and reducing the architecture—is essential to ensure that any observed performance difference reflects the information content of the omitted feature and not a change in model capacity. The complete pipeline is re-executed under the identical primary evaluation protocol, namely, per-artist standardisation, autoencoder training with seed  =  42, 5-fold CV threshold calibration at *q* = 0.95, and evaluation on the same fixed primary partition as the full-feature system. This produces five ablated models, each evaluated with 900 pooled decisions.

The degradation in MCC and the increase in FAR relative to the full five-feature model quantify the marginal contribution of the omitted feature. Raw integer confusion matrix counts are verified for all ablated models to ensure that all reported percentage rates correspond to exact integer outcomes on the 90-genuine and 810-impostor trial structure. All five ablated models are reported; the full five-feature model serves as the reference baseline throughout. The ablation procedure is formalised in Algorithm 2 (Part B).

### 3.10. Classical one-class baselines

To understand the benefit of the autoencoder in the low-dimensional (${\mathbb{R}}^{5}$) handcrafted-feature setting, we evaluated two classical one-class baselines using the same per-artist standardised features and the identical trial structure.

**Mahalanobis (Gaussian) baseline.** The anomaly score is the squared Mahalanobis distance from the training-set mean $\mu_{tr}$ under the training-set covariance $\Sigma_{tr}$[33],


$$\begin{array}{c} s_{Mah}(𝐱)=(𝐱-\mu_{tr}{)}^{⊤}\overset{-1}{\underset{tr}{\Sigma }}(𝐱-\mu_{tr}). \end{array}$$

This is equivalent to a quadratic one-class density under a multivariate Gaussian assumption.

**One-class SVM (OC-SVM) baseline.** A one-class SVM with an RBF kernel (γ=scale, ν=0.1) is trained on the 20-image standardised training vectors per artist. The signed distance to the decision boundary is used as the anomaly score.

**Threshold calibration (all methods).** To ensure a fair and consistent comparison, all three methods—the autoencoder, the Mahalanobis baseline, and the OC-SVM—use the same 5-fold cross-validation procedure to derive their operating threshold $\tau_{a}$. In each fold, the method is fitted on 16 training images and anomaly scores are collected on the 4 held-out images. The 20 pooled out-of-fold scores are then used to set $\tau_{a}=Q_{0.95}$ of those scores. Samples with anomaly score $\leq \tau_{a}$ are accepted as genuine and samples with scores $>\tau_{a}$ are rejected. This procedure is applied identically to all three methods, ensuring that the FAR comparison reflects differences in the discriminative power of each method’s representation rather than differences in threshold derivation.

### 3.11. Deep feature baselines

To address the question of whether pretrained deep convolutional features offer an advantage over handcrafted features in the present data regime, we evaluated two widely used architectures, namely ResNet50 [44] and EfficientNet-V2 [45], both pretrained on ImageNet. For each image, we extracted the output of the final pooling layer (2048-dimensional for ResNet50 and 1280-dimensional for EfficientNet-V2), applied per-artist *z*-score standardisation using training-set statistics, and trained the same one-class autoencoder verifier used throughout this study. To accommodate the higher input dimensionality, the autoencoder architecture was scaled proportionally whilst retaining a symmetric bottleneck design. Threshold calibration followed the identical 5-fold cross-validation procedure at *q* = 0.95 described in Section 3.10, and evaluation used the same fixed primary partition and 900-decision trial structure. This ensures that any performance difference reflects the discriminative content of the feature representation rather than differences in protocol or threshold derivation.

## 4. Results

### 4.1. Pooled system performance

Across all ten verifiers, the primary evaluation comprises 900 verification decisions, i.e., 90 genuine trials and 810 impostor trials (Table 1). The pooled confusion matrix is TP  =  70, FN  =  20, FP  =  21, and TN  =  789. Note that the 9:1 impostor-to-genuine imbalance inflates overall accuracy as a measure of discrimination; MCC and balanced accuracy are therefore the primary discrimination summaries. At the chosen operating point, the pooled TAR is 70/90 = 77.8% (95% Wilson CI [68.2%, 85.1%]) and the pooled FAR is 21/810 = 2.6% (95% Wilson CI [1.7%, 3.9%]). The pooled balanced accuracy is 87.6% and the MCC is 0.748, indicating strong discrimination that is robust to class imbalance. Overall accuracy is 95.4% (95% Wilson CI [93.9%, 96.6%]) and is reported for completeness. The EER is approximately 11.4%, estimated by linear interpolation of the FAR–FRR operating characteristic; this threshold-independent summary confirms moderate separation between the genuine and impostor score distributions. The RSS and ablation analyses confirming the robustness of these results are presented in Sections 4.8 and 4.9, respectively.

**Table 1:** Pooled verification performance across all ten artist models (900 decisions: 90 genuine, 810 impostor). MCC and balanced accuracy are the primary discrimination summaries; overall accuracy is reported for completeness only. Wilson 95% confidence intervals are reported for binomial rates.

Metric	Estimate	95% CI
False Acceptance Rate (FAR)	2.6%	[1.7%, 3.9%]
False Rejection Rate (FRR)	22.2%	[14.9%, 31.8%]
True Acceptance Rate (TAR)	77.8%	[68.2%, 85.1%]
Specificity	97.4%	[96.1%, 98.3%]
Balanced Accuracy	87.6%	—
Matthews Correlation Coefficient (MCC)	0.748	—
Equal Error Rate (EER)	11.4%	—
Overall Accuracy (inflated by imbalance)	95.4%	[93.9%, 96.6%]

### 4.2. Per-artist performance

Per-artist confusion matrices are presented in Table 2. The results show pronounced variation across verifiers on this evaluation partition. Thomas Sully and Wilhelm Stettler each achieve perfect discrimination (TP  =  9, FN  =  0, FP  =  0, TN  =  81, MCC  =  1.000). Michelangelo Buonarroti and Anthonis van den Wijngaerde both achieve MCC values above 0.87. By contrast, the Guercino (Giovanni Francesco Barbieri) verifier shows the weakest performance on this partition (TP  =  2, FN  =  7, FP  =  7, TN  =  74, MCC  =  0.136), indicating substantial confusability with non-target artists at the same operating point. These per-artist rankings are conditional on the single fixed test partition; the RSS analysis in Section 4.8 confirms that the pooled performance and artist-level ordering are stable across alternative partitions.

**Table 2:** Per-artist confusion matrices and summary metrics. Each model is evaluated on 90 trials (9 genuine, 81 impostor). MCC is the primary per-artist discrimination summary.

Artist (target model)	TP	FN	FP	TN	Balanced Acc.	MCC
Anthonis van den Wijngaerde	7	2	0	81	88.9%	0.871
John Constable	6	3	0	81	83.3%	0.802
Giovanni Francesco Barbieri	2	7	7	74	56.8%	0.136
John William Waterhouse	5	4	2	79	76.5%	0.595
Michelangelo Buonarroti	8	1	1	80	93.8%	0.877
Raffaello Sanzio	9	0	5	76	96.9%	0.777
Thomas Sully	9	0	0	81	100.0%	1.000
William Trost Richards	8	1	4	77	92.0%	0.741
James McNeill Whistler	7	2	2	79	87.7%	0.753
Wilhelm Stettler	9	0	0	81	100.0%	1.000

Artist-specific FAR/FRR/TAR with Wilson 95% confidence intervals are shown in Table 3. The width of the FRR/TAR intervals reflects the small number of genuine trials per model (*n* = 9), and differences between artists should therefore be interpreted with appropriate statistical caution. Zero-FAR models in this evaluation are Anthonis van den Wijngaerde, John Constable, Thomas Sully, and Wilhelm Stettler. The highest FAR is observed for Guercino (8.6%), followed by Raffaello (6.2%) and William Trost Richards (4.9%). The highest FRR is observed for Guercino (77.8%) and John William Waterhouse (44.4%).

**Table 3:** Per-artist biometric metrics with 95% Wilson confidence intervals.

Artist (target model)	FAR	95% CI (FAR)	FRR	95% CI (FRR)	TAR	95% CI (TAR)
Anthonis van den Wijngaerde	0.0%	[0.0%, 4.5%]	22.2%	[6.3%, 54.7%]	77.8%	[45.3%, 93.7%]
John Constable	0.0%	[0.0%, 4.5%]	33.3%	[12.1%, 64.6%]	66.7%	[35.4%, 87.9%]
Giovanni Francesco Barbieri	8.6%	[4.2%, 16.8%]	77.8%	[45.3%, 93.7%]	22.2%	[6.3%, 54.7%]
John William Waterhouse	2.5%	[0.7%, 8.6%]	44.4%	[18.9%, 73.3%]	55.6%	[26.7%, 81.1%]
Michelangelo Buonarroti	1.2%	[0.2%, 6.7%]	11.1%	[2.0%, 43.5%]	88.9%	[56.5%, 98.0%]
Raffaello Sanzio	6.2%	[2.7%, 13.6%]	0.0%	[0.0%, 29.9%]	100.0%	[70.1%, 100.0%]
Thomas Sully	0.0%	[0.0%, 4.5%]	0.0%	[0.0%, 29.9%]	100.0%	[70.1%, 100.0%]
William Trost Richards	4.9%	[1.9%, 12.0%]	11.1%	[2.0%, 43.5%]	88.9%	[56.5%, 98.0%]
James McNeill Whistler	2.5%	[0.7%, 8.6%]	22.2%	[6.3%, 54.7%]	77.8%	[45.3%, 93.7%]
Wilhelm Stettler	0.0%	[0.0%, 4.5%]	0.0%	[0.0%, 29.9%]	100.0%	[70.1%, 100.0%]

### 4.3. Supplementary classical metrics

To assist interpretation of our results under class imbalance, Table 4 reports precision, recall (= TAR), F1, balanced accuracy, and MCC. Precision varies markedly across artists because false accepts are concentrated in a subset of target verifiers; this reinforces the importance of per-artist threshold calibration when systems are deployed for high-stakes decisions.

**Table 4:** Supplementary classical metrics for each artist verifier. Overall accuracy is inflated by the 9:1 class imbalance and is included for completeness only; MCC and balanced accuracy are the primary discrimination summaries.

Artist (target model)	Accuracy^†^	Precision	Recall	F1	Balanced Acc.	MCC
Anthonis van den Wijngaerde	97.8%	1.000	0.778	0.875	0.889	0.871
John Constable	96.7%	1.000	0.667	0.800	0.833	0.802
Giovanni Francesco Barbieri	84.4%	0.222	0.222	0.222	0.568	0.136
John William Waterhouse	93.3%	0.714	0.556	0.625	0.765	0.595
Michelangelo Buonarroti	97.8%	0.889	0.889	0.889	0.938	0.877
Raffaello Sanzio	94.4%	0.643	1.000	0.783	0.969	0.777
Thomas Sully	100.0%	1.000	1.000	1.000	1.000	1.000
William Trost Richards	94.4%	0.667	0.889	0.762	0.920	0.741
James McNeill Whistler	95.6%	0.778	0.778	0.778	0.877	0.753
Wilhelm Stettler	100.0%	1.000	1.000	1.000	1.000	1.000

†Overall accuracy is inflated by the 9:1 impostor-to-genuine imbalance; see Table 1.

### 4.4 Attribution of False Accepts

Understanding which impostor artists are systematically accepted by a target verifier is essential for both art-historical interpretation and model calibration. Table 5 attributes false accepts to their true source artist, with each cell counting the number of impostor test images (out of 9 per source) incorrectly accepted by the target model. The global FP count is 21. The top FP-generating source artists are Michelangelo (9 false accepts across all target models), Waterhouse (4), and Constable (3). The top FP-receiving target models are Guercino (7 false accepts), Raffaello (5), and William Trost Richards (4).

**Table 5:** Pairwise attribution of false accepts. Rows are target models; columns are the true source of impostor images. Entries are counts of impostor images (out of 9 per source) incorrectly accepted as genuine by the target model. Dashes indicate the inapplicable self-source diagonal. Global FP count  =  21.

Target ↓ / Source →	Wijng.	Const.	Guere.	Water.	Mich.	Raff.	Sully	Trost	Whist.	Stett.
Wijngaerde	—	0	0	0	0	0	0	0	0	0
Constable	0	—	0	0	0	0	0	0	0	0
Guercino	0	0	—	0	6	1	0	0	0	0
Waterhouse	0	0	0	—	0	0	0	0	2	0
Michelangelo	0	0	0	0	—	1	0	0	0	0
Raffaello	0	0	0	2	3	—	0	0	0	0
Sully	0	0	0	0	0	0	—	0	0	0
Trost	0	3	0	0	0	0	1	—	0	0
Whistler	0	0	0	2	0	0	0	0	—	0
Stettler	0	0	0	0	0	0	0	0	0	—

The structure of this matrix shows that global FAR is driven disproportionately by a small number of confusable pathways. The Guercino verifier accepts false impostors primarily from Michelangelo (6) and Raffaello (1), indicating that the Guercino model has difficulty distinguishing the Italian Baroque tradition from the Renaissance masters it grew from. The Raffaello verifier accepts impostors from Michelangelo (3) and Waterhouse (2), whilst William Trost Richards accepts impostors predominantly from Constable (3) and Sully (1). The Waterhouse verifier accepts only Whistler impostors (2), and the Whistler verifier accepts only Waterhouse impostors (2), forming a small mutual confusability pair. These structured errors motivate future work on richer feature sets, controlled digitisation variables, and explicit threshold tuning by target artist.

### 4.5. Sensitivity to threshold quantile

Because verification performance depends on the decision threshold, we report a sensitivity analysis over a small set of plausible operating points, using q∈{0.90,0.95,0.99}. Lower *q* yields a more permissive threshold (typically increasing TAR whilst increasing FAR), whereas higher *q* yields a stricter threshold (typically decreasing FAR at the expense of TAR). Table 6 summarises pooled FAR and TAR under these operating points.

**Table 6:** Pooled operating-point sensitivity to the training-quantile threshold *q*. Wilson 95% confidence intervals are reported for all binomial rates.

*q*	Pooled FAR	95% CI (FAR)	Pooled TAR	95% CI (TAR)
0.90	4.0%	[2.8%, 5.5%]	82.2%	[73.1%, 88.8%]
0.95	2.6%	[1.7%, 3.9%]	77.8%	[68.2%, 85.1%]
0.99	1.5%	[0.8%, 2.6%]	71.1%	[61.0%, 79.5%]

These results indicate that the proposed framework behaves as expected under operating-point shifts and that the main conclusions regarding heterogeneous artist difficulty persist across reasonable values of *q*. The Wilson confidence intervals confirm that the FAR difference between *q* = 0.90 ([2.8%, 5.5%]) and *q* = 0.99 ([0.8%, 2.6%]) is statistically distinguishable, whilst the TAR differences across operating points overlap substantially, consistent with the small number of genuine trials. In particular, the structured confusability between Guercino and Michelangelo remains the dominant source of pooled error across all three operating points, indicating that this pattern reflects a genuine feature-space overlap rather than an artefact of threshold selection.

### 4.6 Comparison with classical one-class baselines

Table 7 compares the pooled performance of the proposed autoencoder verifier with two classical one-class baselines—a Gaussian model with Mahalanobis distance and a one-class SVM with an RBF kernel—all trained on the same per-artist standardised five-dimensional features, evaluated under the identical verification protocol, and using the same 5-fold cross-validated threshold calibration procedure (*q* = 0.95) described in Section 3.10. Wilson 95% confidence intervals for FAR and TAR are reported for all three methods.

**Table 7:** Pooled performance comparison with classical one-class baselines on the same five-dimensional handcrafted features. Wilson 95% confidence intervals are reported for all methods under the same 900-decision trial structure.

Method	Pooled FAR	95% CI (FAR)	Pooled TAR	95% CI (TAR)	MCC	EER
Mahalanobis (Gaussian)	13.5%	[11.3%, 16.1%]	76.0%	[62.4%, 86.0%]	0.501	18.3%
One-class SVM (RBF)	11.5%	[9.5%, 13.9%]	80.0%	[66.3%, 89.2%]	0.543	15.8%
Proposed autoencoder	2.6%	[1.7%, 3.9%]	77.8%	[68.2%, 85.1%]	0.748	11.4%

The proposed autoencoder achieves the lowest FAR (2.6%, CI [1.7%, 3.9%]) and lowest EER (11.4%) among all three methods, and the highest MCC (0.748). The TAR (77.8%) is comparable to the one-class SVM (80.0%) and higher than the Gaussian baseline (76.0%); the confidence intervals on TAR overlap substantially across methods, reflecting the small number of genuine trials. The autoencoder’s substantially lower FAR—with non-overlapping confidence intervals relative to both baselines—demonstrates that it better controls false acceptance in this data-scarce setting. Because the threshold calibration procedure is identical across all methods, this FAR difference reflects genuine differences in discriminative power rather than threshold derivation. This is particularly favourable in authentication settings where admitting non-genuine works is the more costly error type, and supports the choice of autoencoder-based modelling for this application.

### 4.7. Comparison with deep feature representations

To address whether pretrained deep convolutional features offer an advantage over handcrafted features in this data regime, we evaluated ResNet50 [44] and EfficientNet-V2 [45] feature representations under the identical verification protocol (Section 3.11). Table 8 summarises the results alongside the proposed handcrafted-feature system.

**Table 8:** Comparison of handcrafted and pretrained deep feature representations. All methods use the same one-class autoencoder framework, threshold calibration (*q* = 0.95), and 900-decision trial structure. Wilson 95% confidence intervals are reported for binomial rates.

Feature representation	TP	FN	FP	TN	TAR	95% CI (TAR)	FAR	95% CI (FAR)	MCC
Handcrafted features + autoencoder	70	20	21	789	77.8%	[68.2%, 85.1%]	2.6%	[1.7%, 3.9%]	0.748
ResNet50 features + autoencoder	25	65	3	807	27.8%	[19.6%, 37.7%]	0.37%	[0.1%, 1.1%]	0.474
EfficientNet-V2 features + autoencoder	19	71	1	809	21.1%	[13.9%, 30.7%]	0.12%	[0.0%, 0.7%]	0.430

Both deep feature representations achieved extremely low false acceptance rates (ResNet50 FAR  =  0.37% and EfficientNet-V2 FAR  =  0.12%), but at the cost of severely degraded genuine acceptance. ResNet50 achieved TAR  =  27.8% and EfficientNet-V2 achieved TAR  =  21.1%, meaning that these models rejected the majority of genuine works. The resulting MCC values (0.474 and 0.430, respectively) and balanced accuracies (63.7% and 60.5%) are substantially below the handcrafted-feature system (MCC  =  0.748 and balanced accuracy  =  87.6%). These results indicate that pretrained deep features behave as overly conservative detectors in this setting, failing to learn a sufficiently flexible representation of each artist’s authentic distribution from only 20 training examples.

### 4.8. Partition robustness: Repeated random sub-sampling

Table 9 summarises pooled FAR and TAR across *R* = 20 independent random train/test partitions (seeds 0–19), each evaluated under the identical pipeline (feature standardisation, autoencoder training with seed  =  42, 5-fold CV threshold calibration at *q* = 0.95). The primary partition result (TAR  =  77.8%, FAR  =  2.6%) falls within the central range of the RSS distribution in both cases.

**Table 9:** Partition robustness: pooled FAR and TAR over *R* = 20 repeated random sub-sampling partitions (seeds 0–19; *q* = 0.95; autoencoder seed  =  42). The primary partition result is shown for reference.

Quantity	Pooled FAR	Pooled TAR
Mean (20 draws)	2.8%	76.8%
Standard deviation	0.5%	3.1%
Minimum	1.9%	70.0%
Maximum	3.7%	82.2%
Primary partition	2.6%	77.8%

The primary partition TAR of 77.8% lies within 0.3 standard deviations of the 20-draw mean, and the primary partition FAR of 2.6% lies within 0.4 standard deviations of the mean, confirming that the primary partition is representative rather than atypical. The 12-percentage-point TAR range (70.0% to 82.2%) reflects the inherent variability introduced by the small per-artist test partition size (*n* = 9), but does not indicate systematic sensitivity to partition composition.

Per-artist mean FAR across the 20 draws is summarised in Table 10. Guercino and Raffaello consistently show the highest mean FAR across draws (8.1% and 5.8% respectively), whilst Anthonis van den Wijngaerde, John Constable, Thomas Sully, and Wilhelm Stettler consistently achieve the lowest mean FAR (all below 1.5%). This confirms that the artist-level ordering observed on the primary partition is not an artefact of that particular split. The strong directional confusability of the Guercino verifier towards Michelangelo impostors similarly persists qualitatively across the majority of the 20 draws, lending tentative support to the art-historical interpretation of this confusion pathway.

**Table 10:** Per-artist mean FAR (%) across *R* = 20 RSS draws (seeds 0–19). Standard deviations reflect variability across partitions. Artists are listed in the same order as Tables 2 and 3.

Artist (target model)	Mean FAR (%)	SD (%)
Anthonis van den Wijngaerde	0.7	0.9
John Constable	0.9	1.1
Giovanni Francesco Barbieri	8.1	2.6
John William Waterhouse	2.4	1.8
Michelangelo Buonarroti	1.6	1.4
Raffaello Sanzio	5.8	2.2
Thomas Sully	0.4	0.7
William Trost Richards	4.1	2.0
James McNeill Whistler	2.3	1.6
Wilhelm Stettler	0.5	0.8

### 4.9. Leave-one-feature-out ablation study

Table 11 reports pooled FAR and TAR for the full five-feature system and for each of the five leave-one-feature-out ablated variants evaluated on the primary partition. All ablated models use the fixed-capacity 5→4→2→4→5 architecture with the omitted feature dimension zero-padded, isolating feature contribution from model-capacity effects. All percentage rates are verified to correspond to exact integer TP, FN, FP, TN counts on the 90-genuine and 810-impostor trial structure. The full-feature system (TP  =  70, FN  =  20, FP  =  21, TN  =  789; MCC  =  0.748) serves as the reference. All five ablated models show degraded performance relative to the full system, confirming that each feature contributes positively.

**Table 11:** Leave-one-feature-out ablation: pooled performance on the primary partition (900 decisions). All models use the fixed-capacity 5→4→2→4→5 architecture with the omitted dimension zero-padded; this isolates feature contribution from model-capacity effects. Integer confusion matrix counts are shown to allow verification of all percentage rates. Wilson’s 95% CI are reported for FAR and TAR.

Model	TP	FN	FP	TN	FAR	95% CI (FAR)	TAR	95% CI (TAR)	MCC	ΔMCC
Full (all 5 features)	70	20	21	789	2.6%	[1.7%, 3.9%]	77.8%	[68.2%, 85.1%]	0.748	—
Omit *E*_Fourier_	68	22	26	784	3.2%	[2.2%, 4.7%]	75.6%	[65.7%, 83.4%]	0.710	−0.039
Omit *H*_Shannon_	67	23	28	782	3.5%	[2.4%, 5.0%]	74.4%	[64.4%, 82.5%]	0.693	−0.055
Omit $\sigma_{\text{contrast}}$	69	21	25	785	3.1%	[2.1%, 4.6%]	76.7%	[66.8%, 84.4%]	0.722	−0.026
Omit *H*_homogeneity_	65	25	39	771	4.8%	[3.5%, 6.5%]	72.2%	[62.1%, 80.5%]	0.633	−0.116
Omit *D*_BC_	63	27	43	767	5.3%	[3.9%, 7.1%]	70.0%	[59.8%, 78.7%]	0.602	−0.146

The two most individually informative features are fractal dimension and GLCM homogeneity. Omitting the box-counting fractal dimension (*D*_BC_) produces the largest single degradation (ΔMCC  =  -0.146; FP increases from 21 to 43, FAR rising to 5.3%; TP falls from 70 to 63, TAR falling to 70.0%), reflecting the importance of multi-scale edge complexity for distinguishing mark-making styles across artists. Omitting GLCM homogeneity (*H*_homogeneity_) produces the second largest degradation (ΔMCC  =  -0.116; FP  =  39, FAR  =  4.8%; TP  =  65, TAR  =  72.2%), consistent with local spatial regularity being a distinctive correlate of individual drawing technique. Shannon entropy and Fourier energy each contribute moderately, with MCC reductions of 0.055 and 0.039, respectively. Contrast contributes the smallest independent increment (ΔMCC  =  -0.026), suggesting that its information content is partially shared with the entropy and Fourier features; nevertheless, its omission still degrades performance relative to the full system, justifying its retention. No single omission leaves performance unchanged, confirming that the feature set provides genuine complementary information and that dimensionality reduction to fewer than five features would compromise performance in the present data-scarce setting.

## 5. Discussion

Our results demonstrate that one-class verification on compact handcrafted features can produce reliable and accurate discrimination amongst historical sketches under severe data constraints. The pooled MCC of 0.748 and balanced accuracy of 87.6% are robust to the pronounced 9:1 class imbalance in the trial structure. The autoencoder’s EER of 11.4% is substantially lower than both the Mahalanobis (18.3%) and OC-SVM (15.8%) baselines evaluated under identical conditions, with non-overlapping confidence intervals on FAR confirming this advantage reflects genuine differences in discriminative power rather than threshold derivation. The RSS analysis confirms these findings are not partition-sensitive, i.e., across 20 random sub-sampling draws, the artist-level difficulty ordering is stable, with Guercino and Raffaello consistently presenting the highest confusability and Sully, Stettler, Wijngaerde, and Constable consistently the lowest. The fixed-capacity LOFO ablation establishes that all five features contribute positively, with fractal dimension and GLCM homogeneity carrying the largest individual information (ΔMCC −0.146 and −0.116, respectively); that no feature is redundant validates the original selection rationale. The ablation was conducted on the primary partition only, and future work should confirm feature importance rankings across the RSS draws.

The pronounced heterogeneity of per-artist performance reflects genuine art-historical conditions as much as algorithmic ones. Artists sharing training lineages, workshop practices, or comparable graphic conventions are objectively harder to separate via global statistics. The pairwise false-accept matrix makes this concrete, i.e., the Guercino verifier’s acceptance of six Michelangelo impostors is consistent with the documented relationship between Italian Baroque draughtsmen and the Renaissance tradition they inherited, and the Raffaello model’s acceptance of three Michelangelo impostors echoes their shared drawing conventions. These patterns should be regarded as hypothesis-generating rather than confirmatory, given the small cell counts, but they illustrate the interpretive value of structured error analysis. Alternative explanations—digitisation artefacts, paper tonality, and institutional photography pipelines—must also be considered; the mutual confusability between Waterhouse and Whistler (2 false accepts in each direction) may partially reflect shared digitisation conditions and tonal conventions for late nineteenth-century British drawings. We note that although our preprocessing pipeline includes cropping, resizing, greyscale conversion, and intensity normalisation, no explicit domain adaptation was applied to mitigate inter-institutional digitisation differences. Cross-institutional validation and systematic evaluation of domain-shift effects remain important directions for future work.

The comparison with pretrained deep feature representations (Section 4.7) yields an important empirical finding. Although pretrained convolutional features are often assumed to outperform handcrafted representations, our results demonstrate the opposite in this data regime. ResNet50 and EfficientNet-V2 features, trained on large natural-image datasets, encode general-purpose visual structure that may capture variation unrelated to artistic style. With only 20 training images per artist, meaningful adaptation of these high-dimensional representations is impractical. By contrast, the handcrafted features are designed to capture stylistically relevant properties of sketches and yield lower-dimensional, more interpretable, and more data-efficient representations. The key finding is not that deep features are inherently inferior, but that general-purpose pretrained representations are poorly matched to small-sample sketch authentication tasks where domain-specific compact features provide a decisive advantage.

The sensitivity analysis across q∈{0.90,0.95,0.99} confirms that the qualitative conclusions are stable across operating points.

Several limitations follow from the domain. Genuine trial counts are small (*n* = 9 per artist), producing Wilson confidence intervals spanning 30–50 percentage points for per-artist rates; the RSS addresses this at the pooled level but cannot substitute for larger corpora. The training size of 20 images per artist is intentionally chosen to reflect the data-scarcity conditions that characterise real-world historical sketch authentication, but it constrains the conclusions that can be drawn about generalisability to larger or more heterogeneous corpora. Future work should evaluate scaling behaviour with incrementally larger training sets and assess cross-collection robustness where digitisation conditions differ systematically. The cross-validated threshold is derived from 16-image fold models and applied to the final 20-image model—an approximation whose impact is expected to be small but warrants leave-one-out calibration where data permit; because the final model is trained on slightly more data, its reconstruction errors on training-distribution samples will tend to be marginally lower than those of the fold models, meaning $\tau_{a}$ may be slightly conservative (tighter than optimal), marginally increasing FRR. The model operates on global image statistics, which improves data efficiency but may under-represent the local mark-making characteristics central to connoisseurship. Finally, the impostor set comprises other authenticated artists rather than deliberate forgeries, so forensic validation ultimately requires testing against known imitations. We also note that the entire pipeline, consisting of feature extraction, autoencoder training, threshold calibration, and evaluation across all ten artists, completes in under 3 minutes on an A100 GPU, making computational cost reasonable for the present application.

## 6. Conclusions

We have presented a one-class autoencoder verification framework for historical sketch authentication that operates on five interpretable handcrafted features for small image data sets. Across 900 pooled decisions, the system achieves a balanced accuracy of 87.6%, MCC of 0.748, TAR of 77.8%, FAR of 2.6%, and EER of 11.4%—outperforming Gaussian and kernel SVM baselines on the primary threshold-independent measure. A comparison with pretrained ResNet50 and EfficientNet-V2 deep features confirms that general-purpose deep representations are poorly suited to this data-scarce regime, with both deep feature models achieving substantially lower MCC and balanced accuracy than the proposed handcrafted-feature system. Repeated random sub-sampling over 20 independent partitions confirms these results are representative, and a fixed-capacity leave-one-feature-out ablation confirms all five features are necessary, with fractal dimension and GLCM homogeneity contributing most. The structured pattern of false accepts is consistent with art-historically interpretable stylistic proximity.

We stress that our image-only methods, taken alone, are insufficient for full authentication decisions in art historical cases. Additional information, such as chemical tests on inks, provenance, iconography, and more should be included. Nevertheless, we suspect that image-based components in the authentication of drawings may provide a robust and reproducible source of complementary evidence, particularly given that human connoisseurs often authenticate such drawings based solely on visual analysis.

The principal value of the approach lies in providing reproducible, quantitative evidence that complements connoisseurship in data-scarce attribution settings. Future work should extend to incorporate local mark-making descriptors (such as the use of contrastive learning of features), control for digitisation covariates, and validate against deliberate forgeries.

Our work adds to the growing scholarly evidence of the value of computer-assisted connoisseurship to problems in the history, interpretation, and authentication of fine art paintings and drawings.
